# Predicting Metabolite-Disease Associations Based on Spy Strategy and ABC Algorithm

**DOI:** 10.3389/fmolb.2020.603121

**Published:** 2020-12-03

**Authors:** Xiujuan Lei, Cheng Zhang, Yueyue Wang

**Affiliations:** School of Computer Science, Shaanxi Normal University, Xi’an, China

**Keywords:** metabolites, disease, associations, spy strategy, ABC algorithm

## Abstract

In recent years, latent metabolite-disease associations have been a significant focus in the biomedical domain. And more and more experimental evidence has been adduced that metabolites correlate with the diagnosis of complex human diseases. Several computational methods have been developed to detect potential metabolite-disease associations. In this article, we propose a novel method based on the spy strategy and an artificial bee colony (ABC) algorithm for metabolite-disease association prediction (SSABCMDA). Due to the fact that there are large parts of missing associations in unconfirmed metabolite-disease pairs, spy strategy is adopted to extract reliable negative samples from unconfirmed pairs. Considering the effects of parameters, the ABC algorithm is utilized to optimize parameters. In relevant cross-validation experiments, our method achieves excellent predictive performance. Moreover, three types of case studies are conducted on three common diseases to demonstrate the validity and utility of SSABCMDA method. Relevant experimental results indicate that our method can predict potential associations between metabolites and diseases effectively.

## Introduction

Metabolomics, an important part of systems biology, is a recently and rapidly developed subject following genomics and proteomics, which have entered many fields closely related to human health, such as nutrition and food science, medical development, and, especially, disease diagnosis (Dunn and Ellis, 2005). Accumulating studies have explored the vital roles that metabolites play in the pathogenesis of disease according to changes in the concentration of metabolites. Moreover, the exploration of metabolite–disease associations is meaningful for a deep understanding of the reason a person becomes ill and promotes the diagnosis and treatment of human diseases.

Although many high-throughput metabolomics technologies have been utilized to testify to the metabolite signatures of diseases, which have reached several achievements, such as the Human Metabolome Database (HMDB) (Wishart et al., 2018), unverified metabolite–disease associations are still in the majority. Furthermore, a weakness of experimental determination to identify metabolite-disease associations is that it is extraordinarily laborious and expensive. Accordingly, owing to the high efficiency and reliability of computational approaches (Jiao and Du, 2016; Wu et al., 2020) to identify metabolite–disease associations, they have attracted attention from scientific communities in the relevant field. RWRMDA (Hu et al., 2018), the first method for mining the associations between metabolites and diseases, has made progress in developing computational methods in this field. However, the shortcoming of the method is the lack of disease similarity in the construction of the RWRMDA model. The RLS algorithm, whose core framework is regularized least squares, is used in other prediction areas, such as miRNA-disease associations (Chen and Yan, 2014). However, this algorithm uses single similarities which only use biological information as similarity and the performance of it is not stable.

**FIGURE 1:**
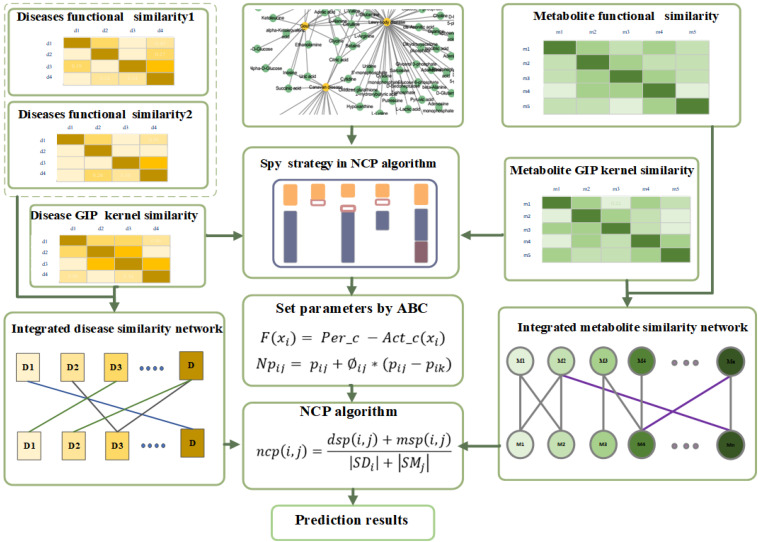
Flowchart of SSABCMDA.

In this article, we put forward a method to predict potential metabolite–disease associations, which utilizes the spy strategy and the artificial bee colony (ABC) algorithm, based on the network consistency projection algorithm (Figure 1). First, we select biological properties of diseases and metabolites and integrate them as biological similarity for diseases or metabolites. Simultaneously, the topological properties of diseases and metabolites are also considered when we calculate the final disease similarity. Second, the spy samples from positive samples are utilized to select latent negative samples with suitable thresholds by spy strategy. Third, the optimized parameters are found by utilizing the ABC algorithm. Finally, the network consistency projection algorithm is used to predict the final scores. The area under the curve (AUC) values of the receiver operating characteristic (ROC) are 0.9412 and 0.9355 (average value) in leave-one-out cross validation (LOOCV) and fivefold cross validation, respectively. The case study of tuberculosis, hepatitis, and asthma deeply showed the effectiveness of our method. In summary, the SSABCMDA method could be a useful and effective algorithm for predicting the metabolite–disease associations.

## Materials and Methods

### Metabolite–Disease Associations

**FIGURE 2:**
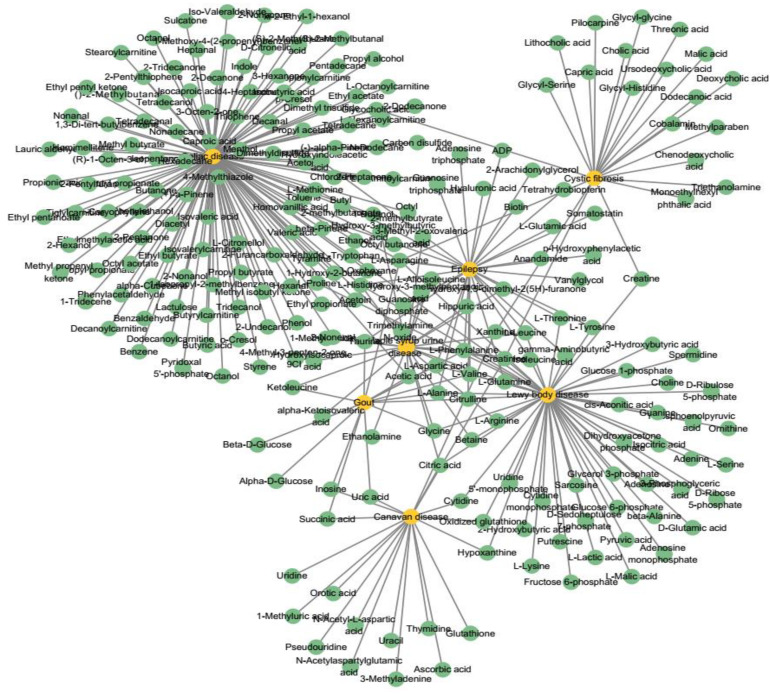
A part of the known metabolite–disease associations network. Yellow nodes represent diseases and green nodes represent metabolites.

The relevant data are extracted from HMDB, DisGeNET (Piñero et al., 2015), and HSDN (Zhou et al., 2014) databases. We firstly extract the disease with DOID and their relevant metabolites in HMDB. Considering integrating the relevant disease similarities, we find the common diseases and their relevant metabolites in DisGeNET and HSDN. Finally, we extract 2,095 experimentally confirmed metabolite–disease pairs, which include 1,401 metabolites and 86 diseases (see Figure 2). The unconfirmed metabolite–disease pairs are regarded as unlabeled pairs. In this study, the number of the investigated metabolites and diseases are defined as variables *nm* and *nd*. To distinctly deliver association information, we establish an adjacency matrix *A* whose size is *nd* rows and *nm* columns. If disease *d*_*i*_ and metabolite *m*_*j*_ are proved to be related, the element *A(i,j)* is set to 1, otherwise 0.

### Disease Functional Similarity 1

The scores of disease functional similarity 1 (DFS1) can be calculated under the hypothesis that two diseases which have more similar features are more likely to be linked with similar genes. The associations of diseases and relevant genes are extracted from DisGeNET (Piñero et al., 2015). Subsequently, the Jaccard similarity is used to calculate similarity score between *d*_*i*_ and *d*_*j*_, which is defined as follows (Gu et al., 2016):

(1) $$DFS1\left(d_{i},d_{j}\right)=\frac{p}{P+q+r}$$

(2) $$G_{n}\left(d_{i}\right)=\left\{ \begin{array}{c} 1,\text{ if }G_{n}\text{ is associated with }d_{i}\text{ and }n\in \left[1,nd\right],\\ 0,\text{ otherwise }, \end{array}\right.$$

where *d*_*i*_ and *d*_*j*_represent two set of diseases. Take *d*_*i*_ as an example, *d*_*i*_ = [*G*_1_(*d*_*i*_),….,*G*_*n*_(*d*_*i*_),..*G*_*nd*_(*d*_*i*_)], and *p* denotes the number of variables with a value of 1 in both *G*_*n*_(*d*_*i*_) and *G*_*n*_(*d*_*j*_) – which means the whole number of genes simultaneously associated with *d*_*i*_ and *d*_*j*_; *q* is defined as the number of variables with a value of 1 in *G*_*n*_(*d*_*i*_) and 0 in *G*_*n*_(*d*_*j*_);and *r* is defined as the number of variables with a value of 0 in *G*_*n*_(*d*_*i*_) and 1 in *G*_*n*_(*d*_*j*_).

### Disease Functional Similarity 2

It is assumed that if two diseases obtain a higher score in a symptom-based similarity matrix, they tend to have a more similar function. We extract the relevant symptoms associated with diseases in HSDN. According to previous articles (Zhou et al., 2014; Ma et al., 2016), every disease has its own set that consists of its relevant symptoms, and disease *i* is taken as an example, which is calculated as follows:

(3) $$D_{i}=\left(w_{i,1},w_{i,2},\ldots ,w_{i,N}\right)$$

(4) $$w_{i,j}=W_{i,j}\log\frac{nd}{nj}$$

where *N* is the total number of symptoms, *w*_*i,j*_ is defined as the weight of the associations between disease *i* and symptom *j*, *nj* denotes the number of diseases that have an association with symptom *j*, *nd* represents the total number of diseases, *W*_*i,j*_ denotes the number of associations of disease *i* and symptom *j*,$\text{log}\frac{nd}{nj}$ could balance the weights problem. Then the disease functional similarity (*DFS2*) between the vectors *D*_*i*_ and *D*_*q*_ of two diseases *i* and *q* is calculated using Equation (5):

(5) $$DFS2\left(d_{i},d_{q}\right)=\cos\left(D_{i},D_{q}\right)=\frac{\sum_{j=1}^{N}D_{i,j}D_{q,j}}{\sqrt{\sum_{j=1}^{N}D_{i,j}^{2}}\sqrt{\sum_{j=1}^{N}D_{q,j}^{2}}}$$

### Metabolite Function Similarity

This is based on the assumption that two metabolites with functional similarity may have more common relevant enzymes. Using a similar way to obtain DFS2, we calculate the weight vector *M*_*a*_of the metabolites, which is the following:

(6) $$M_{a}=\left(w_{a,1},w_{a,2},\ldots ,w_{a,G}\right)$$

(7) $$w_{a,b}=W_{a,b}\log\frac{nm}{nb}$$

where *G* is the number of metabolite-related enzymes, *w*_*a,b*_ quantifies the strength of the associations between metabolite *a* and enzyme *b*, *n*_*b*_ means the number of metabolites associated with enzyme *b*, *nm* represents the total number of metabolites, *W*_*a,b*_ denotes the number of associations between metabolite *a* and enzyme *b*,$\log\frac{nm}{nb}$ could balance the weights problem. Finally, the similarity between the vectors *M*_*a*_ and *M*_*y*_of two metabolites *a* and *y* is calculated as follows:

(8) $$\begin{array}{lll} MFS\left(m_{a},m_{y}\right) & = & \cos\left(M_{a},M_{y}\right)\\ & = & \frac{\sum_{b=1}^{G}M_{a,b}M_{y,b}}{\sqrt{\sum_{b=1}^{G}M_{a,b}^{2}}\sqrt{\sum_{b=1}^{G}M_{y,b}^{2}}} \end{array}$$

### Gaussian Interaction Profile Kernel Similarity

If we consider the hypothesis that similar metabolites tend to reflect a similar pattern of interaction and non-interaction with diseases, the Gaussian interaction profile (*GIP*) kernel similarity for metabolites and diseases based on the topologic information of known metabolite-disease association network is calculated as follows (Wu et al., 2018; Fang and Lei, 2019):

(9) $$KM\left(m_{i},m_{j}\right)=exp\left(-\omega_{m}{∥A\left(:,i\right)-A\left(:,j\right)∥}^{2}\right)$$

(10) $$KD\left(d_{i},d_{j}\right)=exp\left(-\omega_{d}{∥A\left(i,:\right)-A\left(j,:\right)∥}^{2}\right)$$

where ω_*m*_ and ω_*d*_ denote parameters about kernel bandwidth (Yu et al., 2018), which could be obtained by the normalization operation of the original bandwidth parameter $\omega_{m}^{\prime },\omega_{d}^{\prime }$ which are set 1, ω_*m*_,ω_*d*_ are defined as follows (Jiang et al., 2017):

(11) $$\omega_{m}=\omega_{m}^{\prime }/\left(\frac{1}{nm}\sum_{i=1}^{nm}{∥A\left(:,i\right)∥}^{2}\right)$$

(12) $$\omega_{d}=\omega_{d}^{\prime }/\left(\frac{1}{nd}\sum_{i=1}^{nd}{∥A\left(i,:\right)∥}^{2}\right)$$

### Integrated Similarity for Diseases

In this section, we first integrate two disease functional similarities using the disease biological characteristic similarity (*DB*), which consists of two disease functional similarities, is shown as follows:

(13) $$DB\left(d_{i},d_{j}\right)=\left\{ \begin{array}{c} DFS1\left(d_{i},d_{j}\right)\text{ if }DFS2\left(d_{i},d_{j}\right)=0\\ \left(1-\alpha \right)DFS2\left(d_{i},d_{j}\right)+\alpha DFS1\left(d_{i},d_{j}\right)\text{ else } \end{array}\right.$$

Then the biological and topological characteristics of diseases are integrated, as follows:

(14) $$SD\left(d_{i},d_{j}\right)=\left\{ \begin{array}{c} DB\left(d_{i},d_{j}\right)\text{ if }DB\left(d_{i},d_{j}\right)\neq 0\\ \left(1-\beta \right)DB\left(d_{i},d_{j}\right)+\beta KD\left(d_{i},d_{j}\right)\text{ otherwise } \end{array}\right.$$

### Integrated Similarity for Metabolites

The integrated metabolite similarity matrix *SM* consists of metabolite functional similarity and *GIP* kernel similarity. The similarity is defined as below:

(15) $$SM\left(m_{i},m_{j}\right)=\left\{ \begin{array}{c} MFS\left(m_{i},m_{j}\right)\text{ if }MFS\left(m_{i},m_{j}\right)\neq 0\\ \left(1-\gamma \right)MFS\left(m_{i},m_{j}\right)+\gamma KM\left(m_{i},m_{j}\right)\text{ otherwise } \end{array}\right.$$

## Results

### Calculation of Metabolite–Disease Association Prediction Scores

A method named network consistency projection (NCP), which is proposed by Gu et al. (2016) and Bao et al. (2017), is utilized to infer metabolite-disease associations. The main idea for network consistency is that the spatial similarity between metabolite *j* associated metabolites in the metabolite similarity network and disease *i* associated metabolites in the metabolite-disease association network or the spatial similarity between disease *i* associated diseases in the disease similarity network and metabolite *j* associated diseases in the metabolite–disease association network is positively related to the association between disease *i* and metabolite *j*. The potential score between disease *i* and metabolite *j* is positively related to the relevant known associations and the spatial similarity in the disease similarity network or metabolite similarity network. There are three steps in the calculation of the predicted score between disease *i* and metabolite *j* (Gu et al., 2016; Bao et al., 2017):

First, the scores for metabolite space projection are calculated as follows:

(16) $$msp\left(i,j\right)=\frac{A_{i}*SM_{j}}{\left|A_{i}\right|}$$

where *msp*(*i*,*j*) denotes the projection score of *SM*_*j*_on *A*_*i*_, *A*_*i*_represents a vector encoding the associations between disease *i* and all metabolites in the metabolite–disease association network, *SM*_*j*_ is defined as a vector denoting the similarities between metabolite *j*, and all metabolites in the metabolite similarity network, |*A*_*i*_| is the length of vector *A*_*i*_.

Secondly, the projection scores about diseases should be calculated as follows:

(17) $$dsp\left(i,j\right)=\frac{SD_{i}*A_{j}}{\left|A_{j}\right|}$$

where *dsp*(*i*,*j*) denotes the projection score of *DS*_*i*_ on *A*_*j*_, *A*_*j*_ represents a vector encoding the associations between metabolite *j* and all diseases in the metabolite-disease association network, *SD*_*i*_ is defined as a vector denoting the similarities between disease *i* and all diseases in the disease similarity network, and |*A*_*j*_| is the length of vector *A*_*j*_.

Finally, the predicted scores are integrated relevant scores of the metabolite space projection and disease space projection, which is defined as:

(18) $$ncp\left(i,j\right)=\frac{dsp\left(i,j\right)+msp\left(i,j\right)}{\left|SD_{i}\right|+\left|SM_{j}\right|}$$

where *ncp*(*i*,*j*) is the possibility score for disease *i* and metabolite *j*, |*SD*_*i*_| denotes the length of *DS*_*i*_, and |*SM*_*j*_| represents the length of *SM*_*j*_.

### Spy Strategy

**FIGURE 3:**
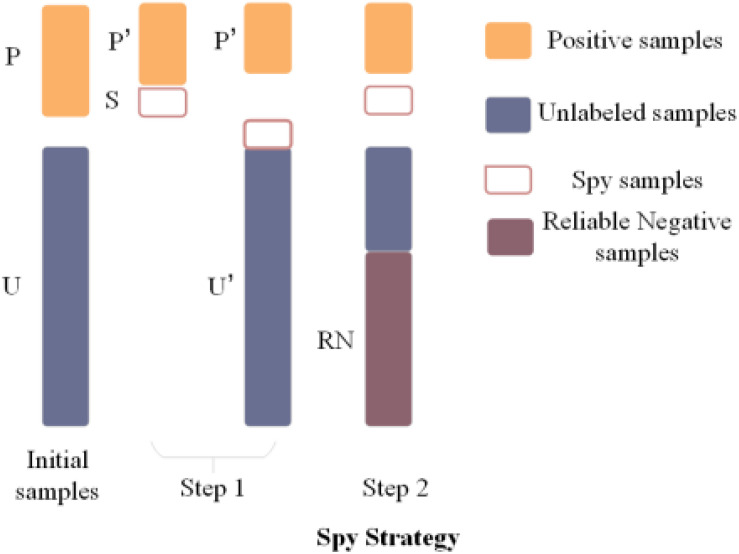
Flowchart of Spy Strategy.

As is generally known, there are many unlabeled metabolite–disease associations in an adjacency matrix, which are regarded as negative training samples most of the time for convenience. But this will cause high false negative rates between predicted associations. Therefore, the spy strategy (Jiang et al., 2017) is utilized to explore the reliable negative samples from the unlabeled metabolite–disease pairs. Spy strategy has several steps. First, 10% spy samples are extracted from the labeled associations, which changes them from 1 to 0. Second, the NCP algorithm and relevant Gaussian kernel similarities are used to get the final score. Then, the score that is the lowest in the spy samples is set to the threshold. If the final score in a candidate sample is lower than the threshold, the relevant value would be set to −1, which is regarded as a reliable negative sample in the association of the metabolite–disease adjacent matrix. Last, the spy samples are repeated 100 times, and the intersection of the reliable negative samples is used as the final reliable negative sample to keep its reliability. The main idea of spy strategy is shown in Figure 3.

### Parameter Analysis Based on ABC

Testing parameters also play a significant role in prediction performance. Moreover, two articles (Wu et al., 2018; Niu et al., 2020) also point out that a swarm intelligence algorithm can optimize parameters and the ABC algorithm (Karaboga and Akay, 2009) is utilized to get the more suitable parameters α, β, and γ in this article. ABC, which is proposed by Karaboga, is inspired by bee colony behavior. In the ABC search process, the algorithm first needs to be initialized, which includes using the number of positions of the honey sources (*nPo*), the maximum number of iterations (*max_iter*), and the range of parameters. Every honey source position can be regarded as a result (parameter set) *x*_*i*_(*i* = 1, 2, 3, 4,., *nPo*) that is a three-dimensional space ranging from 0 to 1. After initialization, the entire population will repeat the search process with employed, onlooker, and scout bees until the *max_iter* is reached. According to the fitness-function (16), all parameter values are tested, and the best parameter values are found at the end of the algorithm. The fitness function *F*(*x*_*i*_) is shown below:

(19) $$F\left(x_{i}\right)=Per\_c-Act\_c\left(x_{i}\right)$$

where *Per*_*c* denotes the perfect and ideal forecast result which is set 1, *Act*_*c*(*x*_*i*_) represents the result about*x*_*i*_, *x*_*i*_ = {α, β, γ and *F*(*x*_*i*_) represents the honey source cost value. The goal is to obtain a set of suitable parameters whose result could make the *F*(*x*_*i*_) turn to be lowest.

**FIGURE 4:**
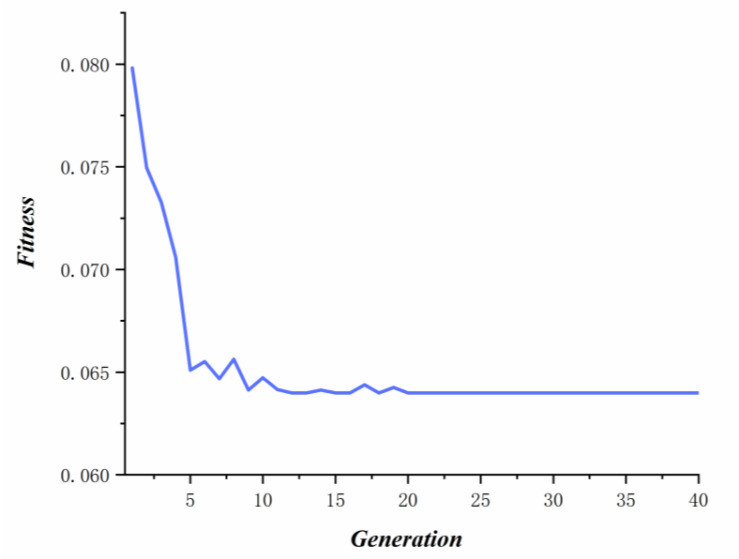
The optimal fitness of each iteration.

At the beginning of the search process, every employed bee finds a new location of honey source by Equation (19):

(20) $$Np_{ij}=p_{ij}+\emptyset_{ij}*\left(p_{ij}-p_{ik}\right)$$

where *k* ∈ [1,*npo*],*j* ∈ [1,*D*] denotes the dimension and *k*≠*i*,∅_*ij*_ ∈ [0,1] is random number. As mentioned above, *x*_*i*_ is a set that consists of the values of parameters α, β, and γ. Thus, *D* is set to 3. After all the employed bees have completed the search, they need to share the relevant information with onlooker bees, and the selection probabilities for each solution are calculated with Equations (20–22):

(21) $$M=\frac{1}{n}*\sum_{i=1}^{n}C_{i}$$

(22) $$F_{i}=e^{-\frac{C_{i}}{M}}i=1,2,\ldots .,n$$

(23) $$P_{i}=\frac{F_{i}}{\sum_{k=1}^{n}F_{i}}i=1,2,\ldots .,n$$

where *n* ∈ [1,*npo*]and*C*_*i*_represents the cost value of the *i*th honey source, and *P*_*i*_ denotes the selecting probability of the *i*th honey source. According to probability of every honey source, on-looker bees select honey source and update relevant honey source. When some honey sources are abandoned, the employed bees corresponded to these sources become scout bees. After the convergence criterion was satisfied, we get best cost value of honey source (see Figure 4) and the optimal parameters (α = 0.56,β = 0.89,γ = 0.6). In this study, *max_iter*, *nPo*, and the number of employed bees are set to 40, 10, and 10, respectively. In addition, the results of *Act*_*c*(*x*_*i*_) is calculated by fivefold cross validation (Luo and Xiao, 2017), where we keep the same division of known associations to reduce the impact of other factors on parameter selection.

### Performance Evaluation

Leave-one-out cross validation (Liu et al., 2019) and fivefold cross validation (Luo and Xiao, 2017) are used as the evaluation tools for our method. For LOOCV, each association that is confirmed in the database is treated as the test sample while the other known associations are viewed as training samples. In addition, those unconfirmed metabolite–disease pairs are regarded as latent candidate samples. For fivefold cross validation, the known metabolite and disease data are randomly split into five equally sized sets. Each set is retained as the validation samples and the other four sets are treated as the training samples. Similar to the LOOCV, the unconfirmed metabolite–disease pairs are used as the candidate samples. Then, the score for each of the validation samples is ranked against the scores of all the candidate samples. At the same time, we obtain the rank for each association in the test samples. To avoid random error caused by the division of known associations, this procedure is repeated 100 times. According to the results of LOOCV and fivefold cross validation, the AUC – the area under the ROC curve – which is calculated from the true positive rate (TPR) and the false positive rate (FPR), is deemed significant. After LOOCV and fivefold cross validation, SSABCMDA yielded the reliable AUC values of 0.9412 and 0.9355 (average value), respectively, which shows that SSABCMDA presents excellent prediction performance.

**FIGURE 5:**
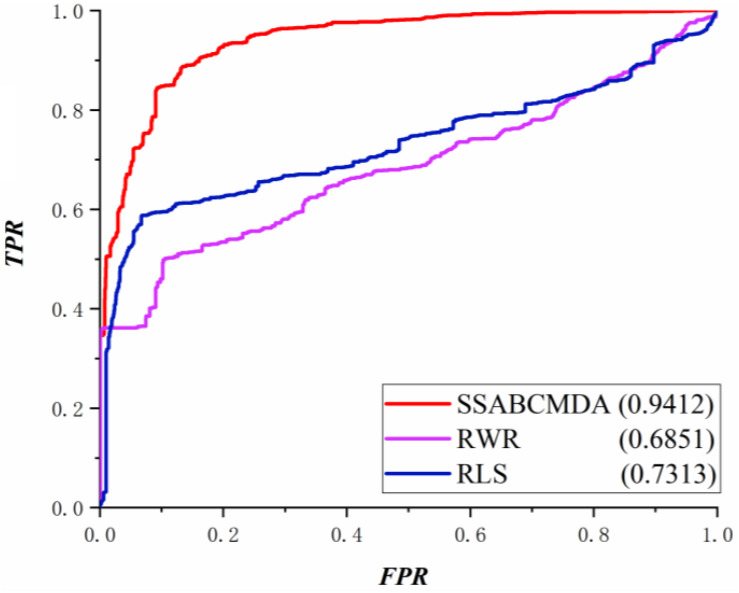
Comparison results about LOOCV.

**FIGURE 6:**
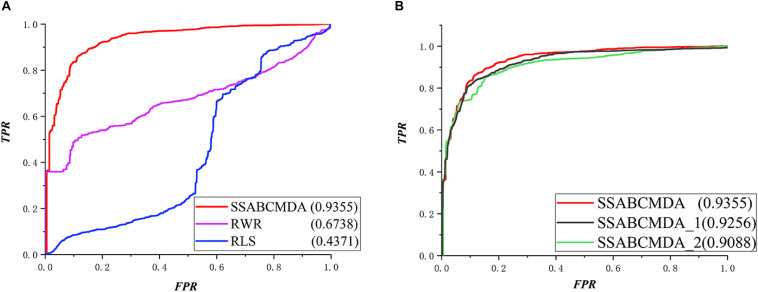
Comparison results about fivefold cross validation. **(A)** Different methods for comparation. **(B)** The combination of different parts in SSABCMDA for comparation.

The RWRMDA (Hu et al., 2018), RLS algorithm is compared with methods based on the same data in this article. The performance comparison in LOOCV is shown in Figure 5, where the AUCs of SSABCMDA, RWRMDA, and the RLS algorithm are 0.9412, 0.6851, 0.7313, respectively. Moreover, SSABCMDA, RWRMDA, and the RLS algorithms gain AUC average values of 0.9355, 0.6738, 0.4371 for fivefold cross validation (see Figure 6A). To explore the effects of spy strategy and ABC algorithm, respectively, we compare SSABCMDA; SSABCMDA_1, which doesn’t consider spy strategy; and SSABCMDA_2, which only uses random parameters. The relevant results for fivefold cross validation are showed in Figure 6B, which indicate that spy strategy and ABC algorithm are effective for predicting performance. As above results showed, we find our method is superior to other methods compared, which indicates that our method is suitable as a reliable biomedical research tool for predicting latent metabolite–disease pairs.

### Case Study

**FIGURE 7:**
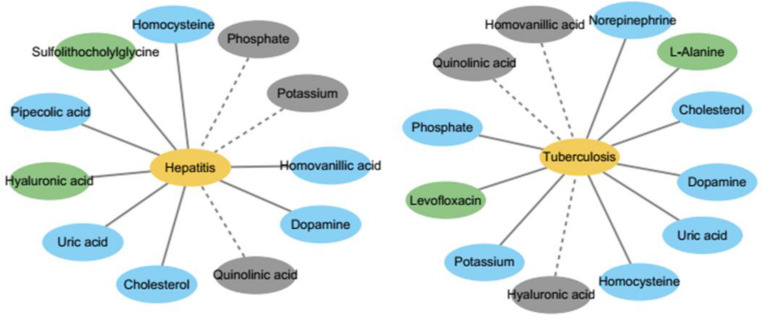
The network of metabolites and diseases. It shows that the top 10 predicted and known metabolites used in this study for two diseases, respectively. The yellow nodes represent diseases and green nodes represent known metabolites which are respective related to two diseases. The blue nodes represent predicted metabolites associated with two disease which are verified by literature, while the gray nodes represent unconfirmed metabolites in top 10 predicted metabolites.

In this section, three diseases – tuberculosis, hepatitis, and asthma – are selected for case studies to explore their pathogenic mechanisms with respect to metabolites. Of the top 10 metabolites predicted, 8, 7, and 7 could be verified from the literature for the three diseases. Two diseases and their known and top 10 predicted metabolites are showed in Figure 7, which is obvious that the confirmed metabolites in top 10 predicted metabolites can help to study the mechanism of disease from the perspective of metabolism.

Hepatitis is the general name for the liver diseases hepatitis A and B. We conducted a case study of Hepatitis on our calculation method. As shown in Table 1, the top 10 metabolites predicted to be interrelated with hepatitis are selected and verified to be correlative. For instance, Uric acid might be useful as a predictive factor for response to therapy for chronic hepatitis (Oh et al., 2017).

**TABLE 1:** Candidate metabolites of hepatitis.

Hepatitis		

Rank	Metabolite name	Evidence
1	Cholesterol	PMID:30600305
2	Uric acid	PMID:28797159
3	Phosphate	- - - - - - - - - - - - -
4	Dopamine	PMID:30386344
5	Homocysteine	PMID:30063074
6	Quinolinic acid	- - - - - - - - - - - - -
7	Homovanillic acid	PMID:4817189
8	Potassium	- - - - - - - - - - - - -
9	Pipecolic acid	PMID:3356409
10	Norepinephrine	PMID:5935605

Tuberculosis is a chronic infectious disease caused by *Mycobacterium tuberculosis*, which can invade the liver and is most common in pulmonary tuberculosis. There are more than eight million new cases of tuberculosis and 1.3 million deaths (Sharma and Mohan, 2004). We carried out a case study of tuberculosis with our method, and 7 out of top 10 metabolites predicted to be interrelated with tuberculosis are verified to be correlative (see Table 2). For instance, the production of NE (norepinephrine) sharply decreased during advanced infection (Barrios-Payán et al., 2016).

**TABLE 2:** Candidate metabolites of tuberculosis.

Tuberculosis		

Rank	Metabolite name	Evidence
1	Cholesterol	PMID:29906645
2	Uric acid	PMID:26398460
3	Phosphate	PMID:27105642
4	Dopamine	PMID:25549893
5	Homocysteine	PMID:28936998
6	Quinolinic acid	- - - - - - - - - - - - -
7	Homovanillic acid	- - - - - - - - - - - - -
8	Hyaluronic acid	- - - - - - - - - - - - -
9	Potassium	PMID:30716121
10	Norepinephrine	PMID:27609282

Asthma is a chronic inflammatory disorder arising from heterogenic gene-environment interactions that are still not fully understood (Mims, 2015). A case study of asthma was carried out with our method, and 8 out of top 10 metabolites predicted had associations with asthma (see Table 3). For example, hyaluronic acid might be a marker of asthma control, as it correlates with airway resistance and has good sensitivity in the detection of impaired asthma control (Kolesov et al., 1968).

**TABLE 3:** Candidate metabolites of asthma.

Asthma		

Rank	Metabolite name	Evidence
1	Cholesterol	PMID:27839668
2	Uric acid	PMID:26509876
3	Phosphate	PMID:26048149
4	Dopamine	PMID:12055141
5	Homocysteine	- - - - - - - - - - - - -
6	Quinolinic acid	PMID:23882022
7	Homovanillic acid	PMID:5717841
8	Hyaluronic acid	PMID:24736408
9	Potassium	PMID:11862989
10	Pipecolic acid	- - - - - - - - - - - - -

## Discussion

In this article, we propose a computational algorithm for metabolite–disease association prediction. To make full use of the information known, we set the known metabolite–disease associations, integrated metabolite similarity, and integrated disease similarity as our input data. The network consistency projection algorithm is utilized as the baseline algorithm. In addition, a spy strategy is utilized to extract negative samples with a high degree of confidence from mixed samples, including potential associations and real negative associations. ABC is introduced to get optimal parameters to improve prediction performance. Moreover, experimental results show reliable evidence that our method is an effective tool to predict metabolite–disease associations. Case studies on three common diseases also give a powerful confirmation to the predictive ability of our method.

The success of our method is due mainly to the following reasons. First, an increasing amount of data known about metabolites and disease has been discovered and confirmed with the development of biological experiments, which are regarded as the basis of predictive data. Second, the network consistency projection as a baseline algorithm guarantees predictive performance. Third, the use of the spy strategy is beneficial to decrease false negative rates of predicted associations. Last, optimal parameters are found quickly with the ABC algorithm, which improves predictive performance.

There are some limitations in the performance of SSABCMDA. At first, although the number of known metabolite–disease associations is larger than before, it is still a small quantity for predictions to obtain sufficiently accurate results. In addition, SSABCMDA depends on the quality of similarity matrices. Some reliable metabolite (disease) similarity matrix from other biological features could be integrated to further expand the algorithm.

## Data Availability Statement

These data about metabolite-disease and metabolite-enzyme associations can be found here: https://hmdb.ca/.

## Author Contributions

XL, CZ, and YW carried out the SSABCMDA method to predict latent associations of metabolites and diseases and participated in its design and drafted the manuscript. All authors read and approved the final manuscript.

## Conflict of Interest

The authors declare that the research was conducted in the absence of any commercial or financial relationships that could be construed as a potential conflict of interest.

## Abbreviations

DAG: Directed Acyclic Graph
GIP: Gaussian interaction profile
LOOCV: Leave-one-out cross validation
TPR: True positive rate
FPR: False positive rate
ROC: Receiver operating characteristic
AUC: Area under the curve.
